# Book and Pamphlet Notices

**Published:** 1859-05

**Authors:** 


### BOOK AND PAMPHLET NOTICES.

I. Manual of Botany. A complete Flora of the Northern States east of the Mississippi, including Virginia and Kentucky. By Asa Gray, Professor of Natural History in Harvard University. With six plates, illustrating the genera of Ferns, etc. P. 606. Published by S. C. Griggs & Co. Chicago, 1859.

II. First Lessons in Botany and Vegetable Physiology. Illustrated by over 360 wood engravings, from original drawings, by Isaac Sprague. By Asa Gray. Chicago, 1859. P. 236. (School and College editions).

These two works constitute but one series; the latter being introductory to the former. They are intended to enable the student, with the aid of a suitable instructor, to acquire a thorough practical knowledge of the science of botany, and to furnish him with such elementary notions of the structure and physiology of plants, as will enable him to understand the uses of their different parts.

From the examination which we have been able to give them, they seem entirely to answer the end for which they are prepared. The "Lessons" are clear, systematic, and illustrated in such a manner, as to be readily comprehended by the youngest beginner in botany. The manual is full, without redundancy, and seems quite complete. It is arranged according to the "natural system" of De Condolle, and has an "Analytical Key to the natural orders, by which the student may refer each plant to its proper family without difficulty."

Prof. Gray's works on botany are standards in many of the higher schools and colleges, and, if any further guarantee of their value be needed, the names of Professors Torrey, Hitchcock, Silliman, Agassiz, and others, furnish all that can be required under this head.

Those who may desire works on this science, whether for schools or private use, cannot do better than to adopt them.

We wish, however, to seize the occasion presented by the notice of these books, and the opening of spring, to say a few words on the value of the study of the vegetable kingdom to all educated persons, as well as to physicians. In a strictly medical point of view, we should have little to say under this head. We have no expectation that any new plant will be discovered, or a new principle in an old one, which will supersede opium, bark, camphor, etc., although it is likely many useful trees are as yet but little known. Neither are we of the belief that the medicinal action of a vegetable substance is better understood, from knowing to what particular genus and species the plant from which it is derived belongs. Having ourselves collected many plants in our student days, and taken doses of nearly all of them, to observe their effects, we may claim to speak from some experience, on that head.

But, in a physiological, hygienic, and æsthetic point of view, there is scarcely a study more useful than that of the vegetable world ; none more pleasing.

1. Physiology.—The cryptogamia are found in such close connection with the infusoria, that it is evident the development of both is favored by the same circumstances. The analogy of the leaves of plants to the lungs of animals is familiar. The following remarks on the movements of plants will be read with interest :

" The stamens of the Barberry, when touched at the base on the inner side, as by an insect seeking for honey, or by the point of a pin, make a sudden jerk forward, and in the process throw some pollen upon the stigma, which stands a little above their reach. In many blossoms the stamens just at the proper season slowly approach the stigma, and after shedding their pollen, recede, or wither away. In such cases, we plainly perceive that a useful end is subserved. But, what shall we say of the Venus' Fly-trap of North Carolina, growing where it is always sure of all the food a plant can need, yet provided with an apparatus for catching insects, and for no other special use that we know of, and actually capturing these expertly by a sudden motion, in the manner already described." By what mechanism is motion effected in organized tissue, where there is no muscular fibre?

We know not, and this is but one instance of numerous inexplicable phenomena connected with the simplest forms of life.

2. As the vegetable furnishes the material by which the animal kingdom is fed, the preservation, increase, and cultivation of plants becomes an object of the first importance.

Commerce, industry, and the useful arts are promoted by every advance in our knowledge of the laws of vegetation.

3. It is the province of vegetation to purify the atmosphere and water by absorbing carbonic acid and other products of animal life, and by giving out oxygen, so that, however prolific the animal creation might be, the vegetable keeps pace with it.

4. Such being the natural relations of animal to vegetable life, it follows that the study of plants is naturally adapted to the faculties and wants of man. The curiosity of the child is drawn toward the various forms of trees, leaves, and flowers, and nothing tends more to give a healthful direction to the thoughts than the encouragement of this interest, by pointing out such facts as are most easily understood, and giving such explanations of familiar things as are suited to the capacity of children. Flowers and plants should be among the attractions of every home, as they tend to keep children from those vagrant habits and unsafe associations from which it is so difficult to preserve them. To love flowers is better than to be precocious, sometimes even better than to be the smartest child in the school. To the grown man, this taste is often a resource against care, anxiety, disappointment, and misfortune; giving a charm, always fresh to the face of nature. To the old it is a precious resource, and a means of retiring gracefully from those pursuits no longer congenial to the latter stage of life. The love and study of plants is to be classed among the hygienic influences of the soul.

Florists and botanists are not often found among the insane; they are charming companions, and safe friends, like the flowers and trees themselves.

There is, in truth, a much more strict alliance between man and trees than the thoughtless might suppose. Fancy, for a moment, the earth without vegetation, and you will feel how much of society there is in the presence of forests. Man at first was at war with all other animals and with his fellows, but found food, shelter and clothing in the vegetable world. Trees, like men, have characters peculiar to each; some are bountiful, and give fruit, useless to themselves, and destined manifestly for the wants of man; others are ornamental, formed to delight the eye. Some are solemn, like the pines which crown the mountain side, lifting their tall heads, and raising their arms to heaven, as in perpetual invocation, while years, generations and centuries pass by.

Some are medicinal, and yield opium and quinine, precious gifts to man. Some are poisonous, and destructive to animals, and to other plants. Emblems of strength, of courage, of glory, of pity, of joy, and of mourning; the oak, the pine, the laurel, the orange, the willow, and the yew, are associated with all the events of man's life, from birth to burial. "Woodman, spare that tree," appeals to the heart and instinctive feelings of man; hence its universal popularity. Nothing fictitious or artificial could ever be so generally felt and understood.

Poets, writers of every class, have always been fond of introducing allusions to flowers into their works. Few of them have done so successfully. Shakspeare and Shelley only knew the use of flowers. Chateaubriand, in his stately prose poetry, speaks of them in words not unfit to be remembered:

"The flower giveth the honey; she is the daughter of the morning, the charm of the spring time, the source of perfumes, the ornament of virgins, the love of poets. She passeth quickly, like the life of man, but sheds her leaves gently upon the earth. Among the ancients, flowers crowned the cup of the banquet, and the white hairs of the sage; the early Christians covered with flowers their altars in the catacombs, and the martyrs of their faith; and even now, in memory of these antique days, we place flowers in our temples."—*Genie du Christianism.*

Finally, the study of flowers is a "medicine for the mind diseased." Men in modern society do not die of old age, nor of disease. Before the fatal attack, anxiety, ambition, disappointment, remorse, and loss of hope, have poisoned the springs of life. It is a consumption of the soul, not of the body; and the good physician, instead of dosing the unfortunate with drugs, should point out to him, and lead him to the contemplation of those natural objects, whose study pleases without agitating, and satisfies without exhausting.

There may be regained, if not too late, that quiet and health, which the too eager pursuit of wealth and station tends to impair and destroy.

Let the young physician, by all means, study the habits and physiology of plants, if he does not become a botanist.

---

III. VENTILATION IN AMERICAN DWELLINGS. With a series of diagrams, presenting examples in different classes of habitations. By DAVID BOSWELL REID, M.D., F.R.S.E., etc. To which is added, an Outline of the Progress of Improvement in Ventilation. By ELISHA HARRIS, M.D., late Physician in chief to the N. Y. Quarantine Hospitals, etc. New York: Wiley & Halsted, 1858. P. 124. From S. C. Griggs & Co., 39 and 41 Lake street, Chicago.

This work is not, strictly speaking, a professional one, and yet a knowledge of the principles and facts which it contains is essential to the physician, as to every one who builds or occupies a house. If you wish to know what is the best way of warming a house, consistent with comfort, health and economy, this is the work to read, or to present to your architect, before he draws your plans.

---

SELECTIONS FROM FAVORITE PRESCRIPTIONS OF AMERICAN PRACTITIONERS. By HORACE GREEN, M.D.

This is a small volume of about 200 pages, made up from selections of favorite prescriptions, which Dr. Green has collected from American physicians.

These formulæ are arranged under the proper heads, and many, no doubt, are useful, and capable of fulfilling the indication for which they are intended.

We would caution the young practitioner, however, from using any particular formulæ, for a certain disease, simply because the diagnosis has been made out, and a certain one recommended.

From S. C. Griggs & Co., 39 and 41 Lake street.

---

FIVE ESSAYS. By JOHN KEARSLEY MITCHELL, M.D., etc. Edited by S. Weir Mitchell, M.D., etc.

The subjects of the different lectures are as follows:

I. Essay on the Cryptogamous Origin of Malasions and Epidemic Fevers.

II. An essay on Animal Magnetism, or Vital Induction.

III. On the Penetsativeness of Fluids.

IV. On the Penetsativeness of Gases.

V. On a new practice in acute and Chronic Rheumatism.

The publication of this volume at this time seems to be rather a mark of respect of a son to a father, than of containing anything new or interesting in medical science, and, therefore, is not a fit work for criticism. The theories advocated in some of the lectures have been set aside by the advance of science, while that of others has been confirmed.

The latter is especially true in regard to the passage of gases and fluids through animal membranes.

To the many friends and former pupils of Professor Mitchell this volume will be eagerly sought after, at least as a memento of their former teacher and friend.

To be had at S. C. Griggs & Co., 39 and 41 Lake street, Chicago.

